# A functional TOC complex contributes to gravity signal transduction in Arabidopsis

**DOI:** 10.3389/fpls.2014.00148

**Published:** 2014-04-22

**Authors:** Allison K. Strohm, Greg A. Barrett-Wilt, Patrick H. Masson

**Affiliations:** ^1^Graduate Program in Cellular and Molecular Biology, Laboratory of Genetics, University of Wisconsin—MadisonMadison, WI, USA; ^2^Mass Spectrometry/Proteomics Facility, University of Wisconsin—MadisonMadison, WI, USA

**Keywords:** gravitropism, roots, TOC complex, plastid, signal transduction, Arabidopsis

## Abstract

Although plastid sedimentation has long been recognized as important for a plant's perception of gravity, it was recently shown that plastids play an additional function in gravitropism. The Translocon at the Outer envelope membrane of Chloroplasts (TOC) complex transports nuclear-encoded proteins into plastids, and a receptor of this complex, Toc132, was previously hypothesized to contribute to gravitropism either by directly functioning as a gravity signal transducer or by indirectly mediating the plastid localization of a gravity signal transducer. Here we show that mutations in multiple genes encoding TOC complex components affect gravitropism in a genetically sensitized background and that the cytoplasmic acidic domain of Toc132 is not required for its involvement in this process. Furthermore, mutations in *TOC132* enhance the gravitropic defect of a mutant whose amyloplasts lack starch. Finally, we show that the levels of several nuclear-encoded root proteins are altered in *toc132* mutants. These data suggest that the TOC complex indirectly mediates gravity signal transduction in Arabidopsis and support the idea that plastids are involved in gravitropism not only through their ability to sediment but also as part of the signal transduction mechanism.

## Introduction

Root gravitropism allows plants to anchor themselves while exploring their environments to gain access to water and nutrients and to avoid obstacles and toxins. In roots, gravity sensing occurs primarily in the columella region of the cap where the cells contain dense, starch-filled amyloplasts that sediment in response to reorientation within the gravity field. Amyloplast sedimentation triggers changes in the localization of plasma membrane-associated auxin efflux facilitators, leading to the accumulation of auxin on the lower side of the root. Upon transmission to the elongation zones, the resulting auxin gradient promotes differential cellular elongation between the upper and lower flanks, resulting in downward curvature. Possible second messengers in this process include Ca^2+^, inositol 1,4,5-triphosphate, and protons. It is still unknown how amyloplast sedimentation leads to an auxin gradient (reviewed in Strohm et al., 2012).

Previously, we showed that ALTERED RESPONSE TO GRAVITY 1 (ARG1) is a peripheral membrane protein that is necessary for a full gravitropic response (Sedbrook et al., 1999; Boonsirichai et al., 2003). *arg1* mutants do not display the characteristic cytoplasmic alkalinization or generate an auxin gradient across the root cap upon reorientation. Expressing *ARG1* in the gravity-sensing cells (statocytes) of only the root or the shoot restores gravitropism only in that organ. Together, these data suggest that ARG1 functions in the statocytes in the early phases of gravity signal transduction. ARG1 localizes to some of the same components of the vesicle trafficking pathway as the PIN auxin efflux carriers and therefore may affect their localization or activity. However, GFP-ARG1 signal is absent from plastids (Boonsirichai et al., 2003). Because *arg1* single mutants display only a partial gravitropic defect and still respond slowly to reorientation, they were used in an enhancer screen. This approach identified *MODIFIERS OF ARG1 1* and *2* (*MAR1* and *2*), which encode components of the Translocon at the Outer envelope membrane of Chloroplasts (TOC) complex (Stanga et al., 2009). Although *mar* single mutants grow normally, *arg1 mar* double mutants show no response to gravity (Stanga et al., 2009).

TOC complexes transport nuclear-encoded proteins into plastids. These complexes consist of a pore (Toc75/MAR1), a Toc159 family receptor (Toc159, Toc132/MAR2, Toc120, or Toc90), and a Toc34 family receptor (Toc33 or Toc34/PPI3). The Toc159 family members contain an N-terminal cytoplasmic acidic domain, a GTP-binding domain, and a C-terminal membrane domain. Most of the variation between these family members occurs within the acidic domain, which has been implicated in substrate selectivity (Inoue et al., 2010). The Toc34/PPI3 members contain only the GTP-binding and membrane domains. Toc132, Toc120, Toc34, and Toc75 are thought to assemble into complexes that tend to import plastid-associated proteins not directly involved in photosynthesis, whereas the import of photosynthesis-related proteins into chloroplasts seems to be mediated mainly by complexes that include Toc159, Toc33, and Toc75 (Ivanova et al., 2004; Kubis et al., 2004).

**Figure 1:**
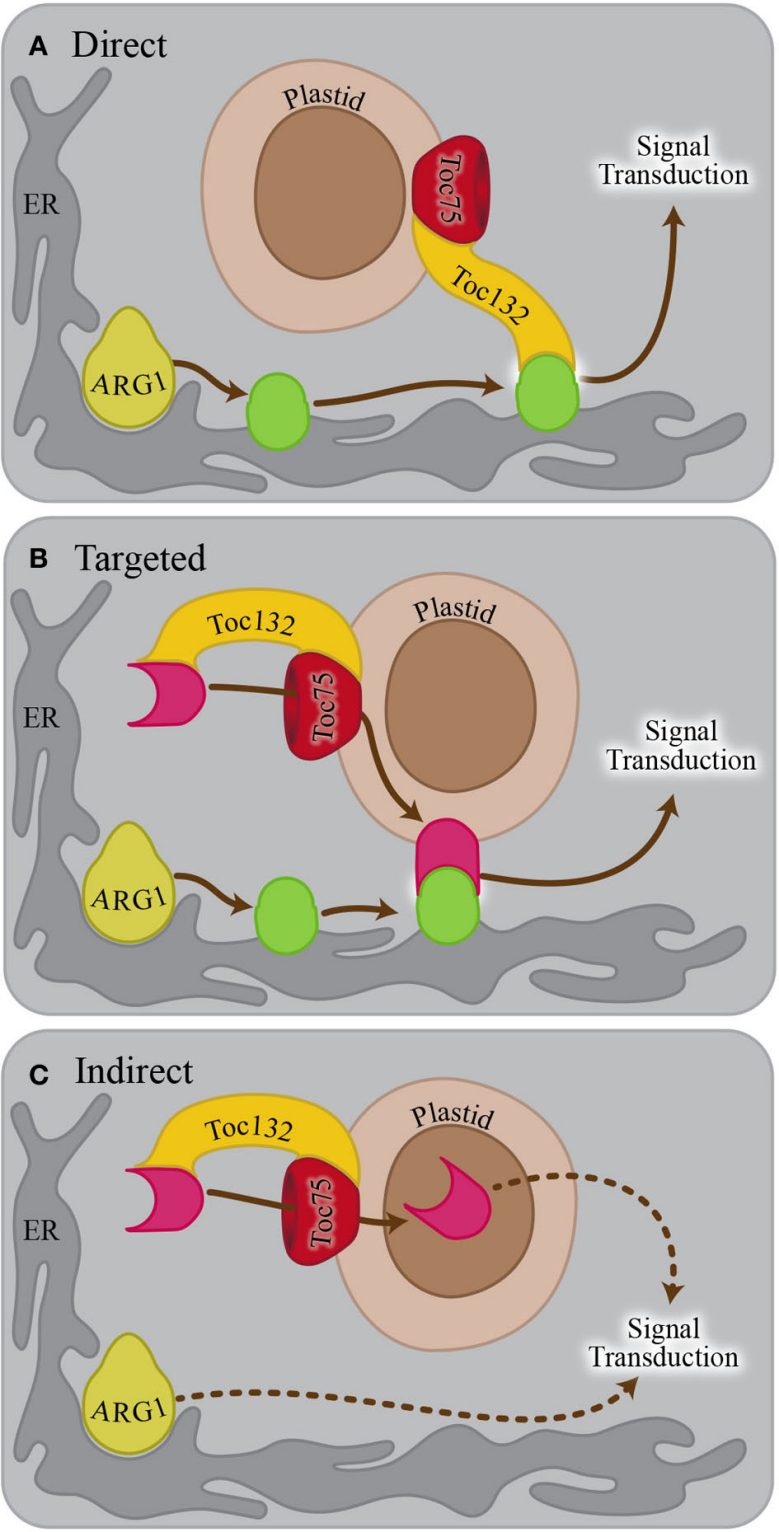
**Possible models explaining the genetic interaction between *ARG1* and *TOC132*. (A)** In the direct interaction model, Toc132 acts as a ligand that interacts with a receptor (green oval) on the ER or plasma membrane. The localization or the activity of the receptor is mediated by ARG1. **(B)** In the targeted interaction model, Toc132 facilitates the plastid localization of a molecule acting as a ligand (pink shape) that interacts with a receptor (green oval) on the ER or plasma membrane upon amyloplast sedimentation. The localization or activity of the receptor is mediated by ARG1. **(C)** In the indirect interaction model, Toc132 facilitates the plastid localization of a molecule that does not act as a ligand but is still required for a gravitropic response in an *arg1* background. In all three panels, the Toc33/34 receptor was omitted from the drawing for the sake of clarity and simplification of the model.

Because ARG1 and the TOC complex localize to different parts of the cell and have different functions, it is not obvious why the corresponding mutations show a strong genetic interaction within the gravitropism signaling pathway. Several hypotheses have been proposed to explain this result (Stanga et al., 2009). In the direct interaction hypothesis, Toc132 directly functions as an amyloplast-associated ligand that interacts with an endoplasmic reticulum (ER)- or plasma membrane-associated receptor upon sedimentation onto these structures (Figure 1A). The genetic interaction between *TOC75* and *ARG1* is consistent with this hypothesis if Toc75 is required for the proper plastid targeting of Toc132. In the targeted interaction hypothesis, the TOC complex mediates the plastid membrane localization of a molecule that interacts as a ligand with a receptor on the ER or plasma membrane (Figure 1B). In the indirect interaction model, the TOC complex facilitates the plastid import of a molecule that does not physically interact with a receptor but is needed for a gravitropic response in an *arg1* background (Figure 1C). The work described here is aimed at testing the direct interaction hypothesis as a first step toward clarifying the role of plastids in the signal transduction cascade between amyloplast sedimentation and auxin redistribution.

## Materials and methods

### Plant materials and growth conditions

*toc120-3* (SALK_017374) and *ppi3-1* were provided by Paul Jarvis (Constan et al., 2004; Kubis et al., 2004), and *toc120-3* was backcrossed to WS wild type before use. *mar2-1* was previously described as a mutation in the *TOC132* gene (AT2G16640) (Stanga et al., 2009). To comply with the Arabidopsis nomenclature, we have renamed this allele *toc132-4*^*mar*2−1^ in this manuscript. The *arg1-2* and *arg1-2 toc132-4*^*mar*2−1^ mutants have also been described previously (Sedbrook et al., 1999; Stanga et al., 2009).

The seeds were sterilized by washing with 95% ethanol. They were plated on half-strength buffered Linsmaier and Skoog medium containing macro- and micro-nutrients, vitamins, and 1.5% sucrose (Caisson Laboratories, North Logan, UT) supplemented with 1.5% agar type E (Sigma-Aldrich, St. Louis, MO) unless otherwise indicated. The seedlings were grown in a Conviron (Asheville, NC) TC16 growth chamber set at 22°C and a 16 h light/8 h dark cycle. The light intensity was 50–70 μmol m^−2^s^−1^ and was provided by cool white fluorescent bulbs (Grainger, Lake Forest, IL).

### Transgenic constructs

The bases encoding the Toc132 GTP-binding and membrane domains (bases 1365–3618 from the start codon) were amplified from Col-0 DNA with the addition of a start codon. This region was cloned in between the AttL1 and AttL2 sites in the Gateway entry vector pENTR/D-TOPO (Life Technologies, Carlsbad, CA). An LR reaction was then performed to transfer this region into the binary vector pMDC32, which placed Toc132GM under the control of the CaMV 35S promoter and the NOS terminator (Curtis and Grossniklaus, 2003; Xu and Li, 2008).

This construct was sequenced and introduced into *arg1-2 toc132-4*^*mar*2−1^ plants using the Agrobacterium-mediated floral dip method (Clough and Bent, 1998). T1 transformants were selected and self-pollinated (Harrison et al., 2006), and T3 seeds likely to carry two copies of the transgene as determined by antibiotic resistance were used.

### Root reorientation kinetics

The seeds were embedded within the medium described above supplemented with 0.7% agar. After at least 2 days of stratification, the seedlings were grown vertically as described above for 8 days. The plates were then turned horizontally, and photographs were taken at select time points. The root tip angle of each seedling at each time point was measured using Adobe Photoshop.

### Protein extraction and mass spectrometry

WS and *toc132-4*^*mar*2−1^ seedlings were grown vertically for 2 weeks on medium supplemented with 0.6% agarose with one genotype grown on medium containing natural abundance ammonium nitrate and potassium nitrate (Sigma-Aldrich) and the other grown on ^15^N-enriched ammonium nitrate and potassium nitrate (Cambridge Isotope Laboratories, Tewksbury, MA) as previously described (Kline et al., 2010; Minkoff et al., 2012). The roots from approximately 300 seedlings per sample were dissected, combined, frozen in liquid nitrogen, and ground using a Mixer Mill 200 (Retsch, Haan, Germany). This process was repeated at non-overlapping times for a total of three trials. For each trial, each genotype was grown on both nitrogen sources for a total of six samples, each containing WS wild-type and *toc132-4*^*mar*2−1^ tissue. Therefore, for each trial, one sample contained WS seedlings grown on natural abundance nitrogen media and *toc132-4*^*mar*2−1^ seedlings grown on ^15^N-enriched media, while the second sample contained *toc132-4*^*mar*2−1^ seedlings grown on natural abundance nitrogen media and WS seedlings grown on ^15^N-enriched media. For each sample and in each trial, the proteins were extracted, trypsin digested, subjected to a solid-phase extraction, and analyzed on an LTQ Orbitrap XL mass spectrometer (Thermo Fisher, Waltham, MA) as previously described (Minkoff et al., 2012). Mascot software v2.2.2 (Perkins et al., 1999) was used to compare the mass spectrometra to sequences present in The Arabidopsis Information Resource protein database (Lamesch et al., 2012). The settings for Mascot searches included permitting up to two missed cleavages. Deamidation of asparagine and glutamine residues and oxidation of methionine residues were set as variable modifications. Cysteines were searched in carbamidomethylated form as a fixed modification. The precursor mass tolerance was set to 20 ppm (allowing for the selection of precursors from the monoisotopic or first or second ^13^C isotopes), and fragment tolerance was set to 0.5 Da. The Mascot output was filtered to a 1.0% false discovery rate using a concatenated decoy database strategy with an in-house written script. Census software (Park et al., 2008) and additional in-house scripts (http://www.biotech.wisc.edu/sussmanlab/research/supporting_Minkoff_2013) were used to make quantitative ratio comparisons between the ^14^N- and ^15^N-labeled signals, and the ratios were normalized to 1 based on the median of each trial. ^14^N/^15^N (light/heavy) ratios were determined for each peptide and were then averaged for all the peptides in each protein. The ^14^N/^15^N ratios of the samples in which WS wild type was labeled with ^14^N were compared to the ^14^N/^15^N ratios of the samples in which WS wild type was labeled with ^15^N using a Student's T-Test and a significance level of *p* < 0.1. For the proteins in which one of these ratios was greater than 1.15 and the other was less than 0.85, the ratio between the genotypes was calculated by taking the inverse of the ^14^N/^15^N ratio for the samples in which *toc132-4*^*mar*2−1^ was labeled with ^15^N and averaging the six *toc132-4*^*mar*2−1^/WS values. The data are available in Data Sheet 1.

### RNA isolation and qRT-PCR

Seedlings were grown as described for the mass spectrometry experiment using medium supplemented with natural abundance ammonium nitrate and potassium nitrate. The roots were dissected with a scalpel, and RNA was prepared using the QIAGEN RNeasy Plant Mini Kit (QIAGEN, Hilden, Germany). The RNA samples were treated with RQ1 DNase (Promega, Madison, WI) according to the instructions. This procedure was conducted at three non-overlapping times for a total of three biological replicates. cDNA synthesis and quantitative real-time PCR (qRT-PCR) were conducted simultaneously using a qScript™ One-Step qRT-PCR Kit (Quanta Biosciences, Gaithersburg, MD) as recommended. Four technical replicates per sample were included. The samples were run on a Roche Light Cycler 480 and analyzed using LinRegPCR (Ramakers et al., 2003). Reference genes with similar expression levels to the candidate genes were chosen (Czechowski et al., 2005); At1g58050 was used for AT4G23690 and GDH3, At4g27960 was used for MAJOR LATEX PROTEIN LIKE 1, and At1g13320 was used for all other genes.

## Results

### Mutations in *TOC120 and TOC34/PPI3* enhance the *arg1* phenotype

**Figure 2:**
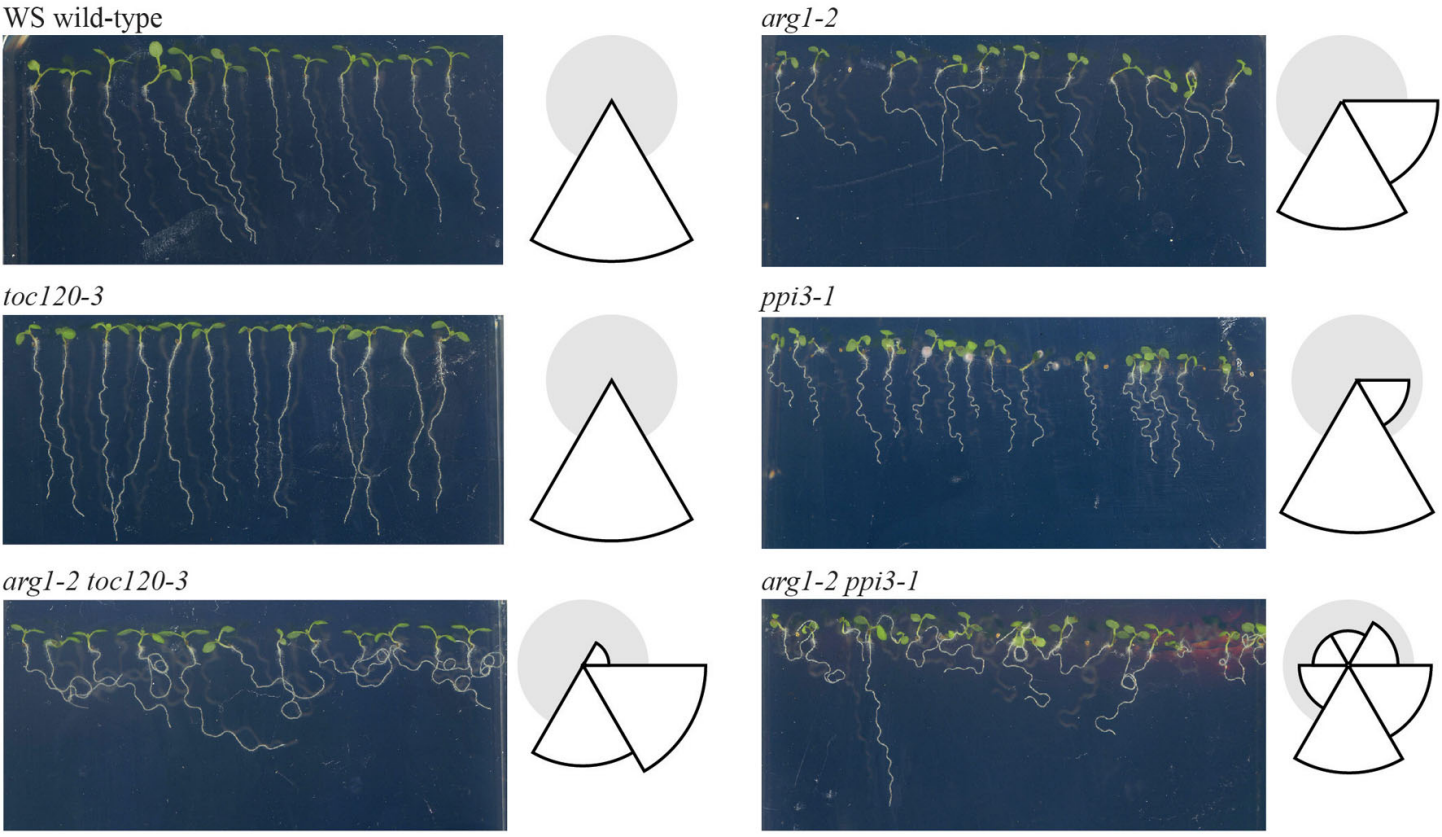
***arg1-2 toc120-3* and *arg1-2 ppi3-1* seedlings showed increased variability of root growth angles compared to WS wild type and *arg1-2*, *toc120-3*, and *ppi3-1* single mutants (*F*-test, *p* < 0.05, and *n* = 32–44).** The seedlings were grown vertically for 3 days. They were then tilted backward 30° and grown for an additional 4 days. The root angles were then measured from the root-hypocotyl junction to the root tip using Adobe Photoshop. These angles were placed into one of six 60° bins, and the corresponding histograms were drawn to the right of each panel. In these histograms, the size of each wedge is proportional to the fraction of plants whose roots grew in the direction of the corresponding bin. This experiment was repeated three independent times with similar results.

If Toc132 specifically acts as a ligand as proposed in the direct interaction model, mutations in other TOC complex receptors are unlikely to enhance the *arg1* gravitropic defect. However, if Toc132 mediates the gravitropic response through its role in plastid protein import, then mutations in other TOC complex receptor genes such as *TOC120* and *TOC34/PPI3* may also enhance the *arg1* mutant phenotype. Therefore, we created *arg1-2 toc120-3* and *arg1-2 ppi3-1* plants and analyzed their gravitropic responses. Although the roots of these double mutants did not grow in completely random directions as did *arg1-2 toc132-4*^*mar*2−1^ and *arg1-2 mar1-1* roots (Stanga et al., 2009), they showed significantly more variable root growth on hard agar surfaces relative to the single mutants (Figure 2). This result suggests that mutations in multiple TOC complex receptors can enhance the *arg1* gravitropic defect, and therefore, protein import via the TOC complex is important for gravitropism. These data indicate that the direct interaction model is unlikely.

### The acidic domain of Toc132 is not required for its role in gravitropism

**Figure 3:**
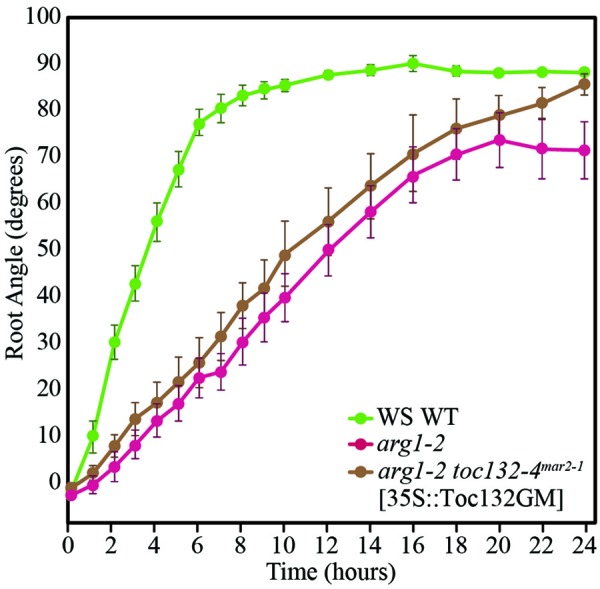
***arg1-2 toc132-4*^*mar*2−1^ [35S::Toc132GM] plants showed similar root tip reorientation kinetics compared to *arg1-2* single mutants but slower kinetics than wild type.** The values in degrees for *arg1-2 toc132-4*^*mar*2−1^ roots, which did not respond to reorientation, for the time points shown on the graph from left to right are −121 ± 29, −120 ± 28, −120 ± 29, −121 ± 29, −120 ± 29, −120 ± 29, −120 ± 29, −120 ± 29, −120 ± 28, −129 ± 29, −120 ± 29, −120 ± 28, −129 ± 28, −129 ± 28, −121 ± 28, −129 ± 29, −121 ± 29, and −120 ± 28. *arg1-2* and *arg1-2 toc132-4*^*mar*2−1^ [35S::Toc132GM] root tip angles were not significantly different from each other at any time point (*p* > 0.05, Student's *T*-Test). The error bars represent the standard error, and *n* = 10. Similar results were obtained in two additional independent experiments.

If the direct interaction model is not supported and Toc132 does not act directly as a ligand, we expect a Toc132 construct that lacks the acidic region but still retains the GTP-binding and membrane domains (Toc132GM) to restore the gravitropic response in an *arg1 toc132* mutant background to that of *arg1* single mutants. Indeed, such a truncated protein is likely to retain its function in protein import into plastids (Inoue et al., 2010) even though it lacks the acidic domain. This domain is the region most likely to act as a ligand because it protrudes into the cytosol and is the most divergent among the TOC complex receptors. We expressed *TOC132GM* in *arg1-2 toc132-4*^*mar*2−1^ mutant plants under the constitutive CaMV 35S promoter and found that the transformed seedlings showed similar root reorientation kinetics to *arg1-2* single mutants (Figure 3). This result demonstrates that the acidic domain of Toc132 is not required for a gravitropic response, and it is consistent with both the targeted and the indirect interaction models.

### Mutations in *TOC132* enhance the *pgm1* phenotype

**Figure 4:**
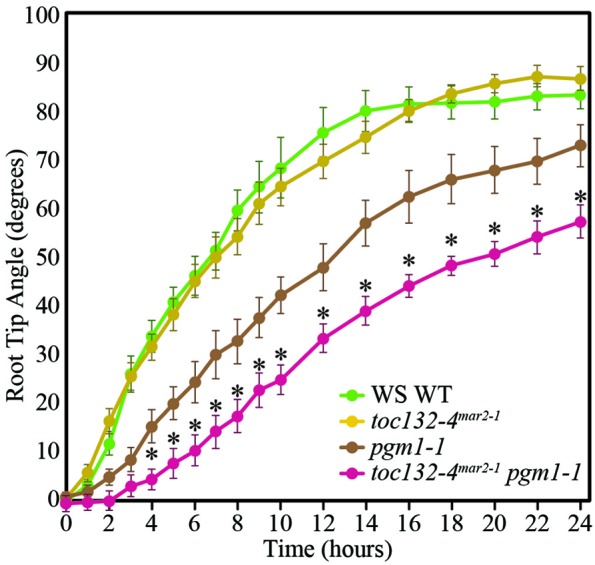
***toc132-4*^*mar*2−1^*pgm1-1* plants showed slower root reorientation kinetics than *toc132-4*^*mar*2−1^ and *pgm1-1* single mutants.** The error bars represent the standard error, and *n* = 10. The asterisks represent significant differences between *pgm1-1 toc132-4*^*mar*2−1^ and the corresponding single mutants (*p* < 0.05, Student's *T*-Test). Similar results were obtained in two additional independent experiments.

We next sought to distinguish between the targeted and indirect interaction models. In the targeted interaction model, the signal transducer imported by the TOC complex triggers signal transduction upon amyloplast sedimentation, whereas the indirect interaction model postulates a role for this plastid-localized transducer that may not rely on amyloplast sedimentation. Therefore, to determine whether the TOC complex functions in conjunction with amyloplast sedimentation, we generated *toc132-4*^*mar*2−1^ *pgm1-1* double mutants. *pgm1-1* single mutants lack starch, and their amyloplasts do not sediment, although they still display some response to gravity (Caspar and Pickard, 1989; Kiss et al., 1989). We found that *toc132-4*^*mar*2−1^ *pgm1-1* double mutants displayed stronger gravitropic defects than *pgm1-1* single mutants (Figure 4). This result is not directly compatible with the targeted interaction model, in which we would expect no enhancement of the gravitropic defect, although it does not rule it out either as the double mutant still retains some gravitropic response.

### The proteome and transcriptome are altered in *toc132* mutants

To identify candidate proteins that might not be properly imported into plastids in *toc132* mutants, we analyzed the wild-type and *toc132-4*^*mar*2−1^ proteomes of whole root tissue. A similar approach was recently used to investigate the levels of plastid-associated proteins using *toc159* whole leaf tissue (Bischof et al., 2011). We expected proteins that were not properly imported into plastids in *toc132* mutants to be degraded or to accumulate and therefore to show differences in expression between the two genotypes. We identified only one protein present at different levels between wild type and *toc132-4*^*mar*2−1^ mutants that was highly likely to localize to plastids (Baginsky and Gruissem, 2009), NUCLEOSIDE DIPHOSPHATE KINASE 3 (NDPK3) (Table 1). However, we found 25 nucleus-encoded proteins present at different levels between wild type and *toc132-4*^*mar*2−1^ that localize to other regions of the cell or whose localizations are unknown. Sixteen of these proteins were more abundant in *toc132-4*^*mar*2−1^, and nine were less abundant (Table 1). Nine of the proteins that were more abundant in *toc132-4*^*mar*2−1^ and seven of those that were less abundant are annotated as functioning in stress responses (Provart and Zhu, 2003).

**Table 1:** **Proteins present at different levels in wild-type and *toc132-4*^*mar*2−1^ roots**.

**Locus**	**Annotation**	***toc132-4^mar2-1^*/ WT ± SE**	***p***
AT2G16005	MD2-related lipid recognition domain-containing protein	2.76 ± 0.49	0.081
AT4G08770	Peroxidase, putative	2.40 ± 0.16	0.019
AT1G54010	Myrosinase-associated protein	2.01 ± 0.07	0.005
AT1G66200	GLUTAMINE SYNTHETASE 1;2 (GLN1;2)	1.76 ± 0.26	0.036
AT1G78320	GLUTATHIONE S-TRANSFERASE TAU 23	1.73 ± 0.24	0.004
AT2G41840	40S Ribosomal protein S2 (RPS2C)	1.60 ± 0.08	0.019
AT4G11290	Peroxidase, putative	1.60 ± 0.26	0.027
AT2G01520	MAJOR LATEX PROTEIN LIKE 1	1.57 ± 0.12	0.034
AT4G22380	Ribosomal protein	1.46 ± 0.10	0.043
AT1G74020	STRICTOSIDINE SYNTHASE 2	1.45 ± 0.09	0.094
AT1G17170	GLUTATHIONE S-TRANSFERASE TAU 24	1.45 ± 0.14	0.052
AT1G66270	BETA-GLUCOSIDASE 21	1.44 ± 0.06	0.010
AT2G38390	Peroxidase, putative	1.43 ± 0.14	0.052
AT3G13930	Dihydrolipoamide S-acetyltransferase, putative	1.43 ± 0.18	0.089
AT1G45145	THIOREDOXIN H-TYPE 5	1.40 ± 0.12	0.094
AT1G19570	DEHYDROASCORBATE REDUCTASE	1.33 ± 0.08	0.030
AT2G36580	Pyruvate kinase, putative	1.22 ± 0.07	0.055
AT3G56070	ROTAMASE CYCLOPHILIN 2	0.84 ± 0.02	0.002
AT1G47128	RESPONSIVE TO DEHYDRATION 21	0.80 ± 0.07	0.100
AT2G38380	PEROXIDASE 22	0.79 ± 0.04	0.058
AT4G11010	NUCLEOSIDE DIPHOSPHATE KINASE 3	0.76 ± 0.02	0.003
AT5G07440	GLUTAMATE DEHYDROGENASE 2	0.72 ± 0.03	0.0003
AT4G23690	Disease resistance-responsive family protein	0.71 ± 0.11	0.063
AT3G03910	GLUTAMATE DEHYDROGENASE 3	0.67 ± 0.04	0.015
AT4G23670	MAJOR LATEX PROTEIN LIKE 6	0.52 ± 0.12	0.025
AT1G20450	EARLY RESPONSE TO DEHYDRATION 10	0.49 ± 0.08	0.079

The annotations were obtained from The Arabidopsis Information Resource. The ratios between the genotypes and the associated standard errors were determined as described in the Materials and Methods section, and a Student's T-test was used to generate the p-values.

**Figure 5:**
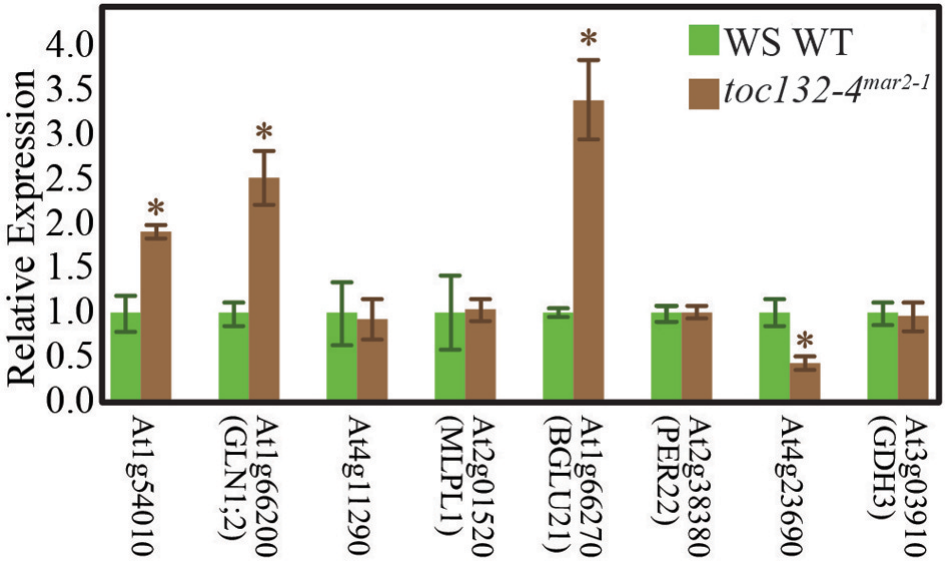
**Many genes whose protein levels are altered in *toc132-4*^*mar*2−1^ also show different transcript levels in *toc132-4*^*mar*2−1^ relative to wild type.** The first five genes from left to right encode proteins that are found at an increased abundance in *toc132-4*^*mar*2−1^, and the last three genes encode proteins that are found at decreased abundance in *toc132-4*^*mar*2−1^. The expression of each candidate gene was quantified relative to a reference gene as described in the Materials and Methods section, and the expression of each gene in wild type was set to 1. The error bars represent the standard error among three biological replicates. The asterisks indicate significant differences between wild type and *toc132-4*^*mar*2−1^ at *p* < 0.05 (Student's *T*-Test).

We then used qRT-PCR to determine if some of the genes encoding these proteins are differentially expressed in *toc132-4*^*mar*2−1^ compared to wild type. The primers are shown in Table 2. Of the eight genes selected for analysis, four showed differences at the transcript level in the same directions predicted by the proteomic analysis (Figure 5).

**Table 2:** **Primers used to quantify gene expression**.

**Locus**	**Forward primer**	**Reverse primer**
AT1G54010	atcccaacggcaaattctc	cgatcggaatcctcatgaat
AT1G66200	cagctgctgatgaaatatggat	aaccacaccagcaatctctgt
AT4G11290	caacatataaacaatgcaccctct	caagatggaaccatcacaacc
AT2G01520	cgacggtgaatgggactc	ccttgaacacctccggttt
AT1G66270	ccaactgttagccgtgtacttg	tttgtggataatctcccgaagt
AT2G38380	tcgtcgatgaactgcagact	gatcgatgcatcacaaccac
AT4G23690	ggcgacaatgtagcaaacg	acttgaagtttcctagtcctggag
AT3G03910	ggaaccggtcctcagaca	aagatcaatgggttttccagtg
AT1G58050	ccattctactttttggcggct	tcaatggtaactgatccactctgatg
AT1G13320	taacgtggccaaaatgatgc	gttctccacaaccgcttggt
AT4G27960	catcttgaaggagcagtggag	gggtttggatccgttaacaa

The sequences are shown 5' to 3'.

## Discussion

We demonstrated that mutations in multiple TOC complex components caused enhanced gravitropic defects in an *arg1* background (Figure 2). This result supports a model in which the TOC complex imports a molecule into plastids that is necessary for gravitropism (Figures 1B,C). However, we did not see completely random root growth when we mutated *TOC120* or *TOC34* in an *arg1* background. This result may have occurred due to the abilities of other receptors such as Toc132 and Toc33 to partially compensate for the loss of these proteins (Kubis et al., 2003, 2004; Ivanova et al., 2004). In any case, these results indicate that Toc132 is unlikely to function as a ligand in gravity signal transduction. This result is in contrast to our previously published result that mutations in *TOC120* do not enhance the *arg1-2* phenotype (Stanga et al., 2009). A closer examination using more seedlings revealed this subtle but consistent phenotype.

Toc159 family members differ most significantly in the lengths and sequences of their N-terminal acidic domains. Although the acidic domain is thought to help regulate the specificity of imported proteins, it has little effect on overall import capacity (Inoue et al., 2010). We showed that a truncated version of Toc132 that lacks the cytoplasmic acidic domain is capable of restoring the gravitropism response of *arg1-2 toc132-4*^*mar*2−1^ seedlings back to that of *arg1-2*. Therefore, we conclude that the acidic domain is not required for Toc132's function in the gravitropic response (Figure 3). Because this construct still contained the GTP-binding and membrane domains, it likely retained most or all of the protein-import capability associated with full-length Toc132 (Inoue et al., 2010). We conclude that this construct likely rescued the *arg1-2 toc132-4*^*mar*2−1^ phenotype because it increased the overall protein import efficiency of the TOC complex. Therefore, Toc132 likely does not directly act as a ligand in gravity signal transduction because the large cytoplasmic acidic domain, which is the most likely region of the protein to interact with a receptor, is not necessary for its gravitropic function. This result reinforces the hypothesis that the TOC complex mediates gravitropism by modulating the targeting to plastids of another important molecule that contributes to gravity signal transduction (Figures 1B,C).

To further test the targeting and indirect models (Figures 1B,C), we examined the gravitropic response of *toc132-4*^*mar*2−1^ *pgm1-1* plants and found that they displayed slightly enhanced gravitropic defects compared to the *pgm1-1* single mutant (Figure 4). This result suggests that *TOC132* and *PGM1* function in different genetic pathways and that the TOC complex contributes to gravitropism in a manner at least partially independent of amyloplast sedimentation. Therefore, the indirect interaction model is plausible (Figure 1C); however, we cannot rule out the targeted interaction model (Figure 1B).

In our analysis of the *toc132-4*^*mar*2−1^ root proteome, we expected many plastid-localized proteins to be differentially expressed between *toc132-4*^*mar*2−1^ and wild type, as previously shown for *toc159* leaf proteins (Bischof et al., 2011). However, we identified only one protein likely to localize to plastids, NDPK3, in a group of 26 differentially expressed proteins between *toc132-4*^*mar*2−1^ and wild type. This protein has also been shown to localize to mitochondria (Sweetlove et al., 2001). NDPKs have been implicated in stress and light signaling, and the related protein NDPK2 has been shown to be involved in auxin-related processes at least partly by affecting auxin transport (Choi et al., 2005). Furthermore, *NDPK3* was previously found to be redox regulated, and redox signals have been implicated in plastid-to-nucleus retrograde signaling (Fey et al., 2005). Therefore, addressing a possible role for NDPK3 in gravitropism is an interesting area of future research.

The low number of plastid-localized differentially expressed proteins may be due to compensation by Toc120 or other receptors. However, we did identify many nucleus-encoded proteins present at different levels in *toc132-4*^*mar*2−1^ relative to wild type, and several of the corresponding genes also showed differences at the transcript level (Figure 5). Plastids can regulate nuclear gene expression through retrograde signaling, especially when they are stressed (Nott et al., 2006). Indeed, many of the proteins present at different levels in *toc132-4*^*mar*2−1^ are involved in stress responses. Alternatively, it is also possible that some of the expression differences we observed between mutant and wild-type roots are indirect consequences of altered cell metabolism in the mutant. Such effects could occur at the transcriptional, posttranscriptional, translational, and posttranslational levels, potentially explaining why several of the differentially expressed proteins are encoded by genes whose transcript levels are similar between mutant and wild-type roots (Table 1 and Figure 5). In any case, our results suggest the possibility that altered plastid protein import results in the altered abundance of (a) nuclear-encoded protein(s) that is (are) required for a partial gravitropic response in an *arg1* background. Interestingly, the genes encoding two of the proteins in Table 1 (MD-2-related lipid recognition domain-containing protein and MAJOR LATEX PROTEIN LIKE 6) increase in expression in root tips upon gravistimulation (Kimbrough et al., 2004).

Considered together, these experiments suggest that the direct interaction model is highly unlikely to explain the role of the TOC complex in gravitropism. Future work will determine which proteins must be imported into plastids for a normal gravitropic response in an *arg1* background and which, if any, non-plastid-associated proteins whose abundance is consequently altered are involved in this process.

## Author contributions

Allison K. Strohm designed and performed the experiments and wrote the paper. Greg A. Barrett-Wilt performed the experiments and revised the paper. Patrick H. Masson designed the experiments and wrote the paper.

## Conflict of interest statement

The authors declare that the research was conducted in the absence of any commercial or financial relationships that could be construed as a potential conflict of interest.
